# First study on genetic variability of bovine viral diarrhea virus isolated from Sapera dairy goats with reproductive disorders in Yogyakarta, Indonesia

**DOI:** 10.14202/vetworld.2022.1015-1021

**Published:** 2022-04-22

**Authors:** N. Retno, H. Wuryastuty, R. Wasito, S. H. Irianingsih

**Affiliations:** 1 Doctoral Study Program, Faculty of Veterinary Medicine, Gadjah Mada University, Yogyakarta, Indonesia; 2 Department of Veterinary Internal Medicine, Faculty of Veterinary Medicine, Gadjah Mada University, Yogyakarta, Indonesia; 3 Department of Veterinary Pathology, Faculty of Veterinary Medicine, Gadjah Mada University, Yogyakarta, Indonesia; 4 Disease Investigation Center, Wates, Yogyakarta, Indonesia

**Keywords:** 5’untranslated region, bovine viral diarrhea virus, reproductive disorder, Sapera dairy goats

## Abstract

**Background and Aim:**

Bovine viral diarrhea (BVD) virus (BVDV) is an important viral pathogen of cattle that can infect diverse artiodactyl species. The clinical manifestations caused by BVDV in heterologous hosts, as they do in cattle, vary, although respiratory and reproductive failures are commonly reported. BVDV infections commonly result in reproductive failure in goats, with abortion being the primary clinical sign. In central Java, Indonesia, BVDV infection has been reported in two clinically healthy local goat species, and the testing indicated infection by BVDV Type 1. However, the genetic diversity of viruses has not been described in healthy or ill goats. The objectives of the present study were as follows: (1) To investigate the genetic variation of BVDV isolated from Sapera dairy goats with naturally occurring reproductive disorders in Yogyakarta, Indonesia, using the 5’ untranslated region (5’ UTR) and (2) to study the possible correlation between reproductive disorders and the presence of BVDV in the flock.

**Materials and Methods:**

Blood samples were collected in October 2021 from 39 goats that had been individually reported to have reproductive disorders. The serum samples were subjected to molecular detection and genetic characterization of BVDV based on the 5’ UTR of the viral genome, followed by sequencing and phylogenetic analyses. Viral isolation was performed on BVDV-positive samples to analyze the viral biotypes.

**Results:**

BVDV infection was detected in five out of 39 female goats. The clinical status of the BVDV-infected goats was abortion (n=2), metritis (n=1), and repeated breeding (n=2). All antigen-positive samples were confirmed as BVDV type 1a (BVDV-1a) and noncytopathic (NCP)-BVDV biotype.

**Conclusion:**

The BVDV-1a and NCP biotypes are the main subtypes and biotypes present in Sapera dairy goats exhibiting reproductive failure. This result is consistent with previous results in dairy cattle in Yogyakarta. The reported results can facilitate the design of methods for the prevention and control of BVD circulating in Indonesia.

## Introduction

Goats are multipurpose animals; their meat, milk, and skin are used for the production of commodities and comprise many domestic products. Reproductive efficiency is one of the essential components of successful goat husbandry. Factors that induce poor reproductive and production performance by causing infertility and affecting the general health status are major concerns due to their economic consequences.

Bovine viral diarrhea (BVD) is a contagious viral disease that significantly affects cattle; therefore, it has a substantial economic impact on the cattle industry worldwide. The disease is caused by BVD virus (BVDV), a positive-sense single-stranded ribonucleic acid (RNA) virus belonging to the genus *Pestivirus* within the family of *Flaviviridae* [1,2]. *Pestivirus* consists of four species: BVDV type 1 (BVDV-1, *Pestivirus* A), BVDV type 2 (BVDV-2, *Pestivirus* B), classical swine fever virus (*Pestivirus* C), and border disease virus (*Pestivirus* D) [3,4]. In recent years, several studies have described new viruses that may represent additional members of the genus *Pestivirus* [5,6]. On the basis of nucleotide sequence comparisons, 21 BVDV-1 subgenotypes (BVDV-1a to BVDV1u) and four BVDV-2 subgenotypes (BVDV-2a to BVDV2d) have been identified [4]. In addition to genotype, BVDV is classified using one of two biotypes, cytopathic (CP) or non-CP (NCP), according to the effect on cells *in vitro* [5]. The NCP-BVDV biotype has been shown to be predominant in the field.

Various clinical manifestations of BVDV infection, from subclinical to fatal disease, have been reported in cattle. Pathogenicity, however, is not limited to cattle, and more than 40 other artiodactyl species can be infected [7,8]. The consequences of BVDV infection in heterologous hosts vary depending on the host- and virus-associated factors such as biotype, genotype, or antigenic variation. However, they are generally similar to those in cattle [9-11]. The typical clinical symptom of BVDV infection in goats is reproductive failure [7,11,12]. In the previous studies, natural BVDV infection in healthy goats was rarely reported, and the goats rarely became reinfected after recovery from infection. Therefore, BVDV in goats was assumed not to be a problem and therefore did not require systematic control [13-15]. However, there is increasing serological evidence of *Pestivirus* infection in goats in many countries, including Indonesia [9,10,16,17], and BVDV-1a, BVDV-1b, and BVDV-2a have been reported in experimentally inoculated goats [9,11,18].

In Indonesia, BVDV infection was first detected in clinically healthy local goats [16], but the genetic variability of BVDV has not been determined or studied. The purposes of this study were (1) to study the biotypic and genotypic variation of BVDV isolated from Sapera female dairy goats with naturally occurring reproductive disorders in Yogyakarta, Indonesia, based on the sequence of the 5’ untranslated region (5’ UTR) and (2) to study the possible correlation between reproductive disorders and the presence of BVDV in flocks.

## Materials and Methods

### Ethical approval

The present study was performed after approval from the Ethical Research Committee, Faculty of Veterinary Medicine, Gadjah Mada University, Yogyakarta, Indonesia (No. 00144/EC-FKH/Int./2021).

### Study period and location

The study was conducted in October 2021. Adult Sapera dairy goats at least 2 years of age that were locally raised and belonged to several smallholder farmers (5-10 heads per farmer) in Turi Village, Sleman, Yogyakarta, Indonesia were selected as experimental animals.

### Sample collection

Adult female Sapera breed dairy goats belonging to several smallholder farmers (5-10 heads per farmer) in Turi Village, Sleman, Yogyakarta, Indonesia. A total of 39 blood samples were collected in October 2021 from four different flocks with reproductive problems. Blood sampling was carried out for each diseased goat only once during the study. Approximately 3 mL of blood samples were taken from the jugular vein using plain vacutainer tubes (Beckton Dickensen). Whole blood samples were processed to collect the sera by centrifuging at 1500× *g* for 15 min at the Veterinary Internal Medicine Laboratory, Faculty of Veterinary Medicine, Gadjah Mada University. The sera samples were then subjected to molecular detection and genetic characterization of BVDV based on the 5’ UTR of the viral genome.

### RNA extraction and reverse transcription (RT) amplification

*Pestivirus* RNA was extracted from sera using a commercially available kit (Viral Nucleic Acid Extraction Kit II, Geneaid Biotech Ltd., Taiwan) following the manufacturer’s instructions. The extracted RNA was subjected to RT and polymerase chain reaction (PCR) amplification in one-step reactions using the MyTaq™ One-Step RT-PCR kit (Bioline Meridian Bioscience, Australia) according to the manufacturer’s instructions with a Biometra Personal Combi thermocycler (37079 Gottingen, Germany). PCR assays were performed to detect general *Pestivirus* using pan-*Pestivirus* generic primers (324F: 5’ ATG CCC WTA GTA GGA CTA GCA 3’ and 326R: 5’ TCA ACT CCA TGT GCC ATG TAC 3’) for amplification of the corresponding gene target of the 5’ UTR [19]. The expected size of the PCR amplification products was 288 bp.

The thermal cycling conditions were as follows: 30 min of RT at 60°C followed by 2 min of initial denaturation at 94°C and 35 cycles of denaturation at 94°C for 30 s, annealing at 55°C for 30 s, and elongation at 72°C for 45 s, with a final extension step at 72°C for 7 min. BVDV genotyping was conducted using nested PCR as previously described [20]. BVDV-1 strain singer was used as a positive control, and sterile distilled water was used in place of template DNA for the negative control reaction.

Following PCR amplification, the products were electrophoresed on a 1.5% agarose gel at 120 V and 400 A for 45 min. The agarose gel was then immersed in a 1× buffer Tris-borate- Ethylenediaminetetraacetic acid™ buffer (Sigma-Aldrich, St. Louis, USA) with 0.5 mg/mL of ethidium bromide (Sigma-Aldrich USA) solution for 15 min. The stained electrophoresed PCR product was then visualized using an ultraviolet transilluminator and imaged using the Gel Logic 100 imaging system (Kodak, Carestream Health, Inc. Rochester, NY 14608).

### Sequencing and phylogenetic analysis

The forward and reverse sequences for each sample were aligned and used in the phylogenetic analysis. The sequences were compared with other previously published sequences. The comparison of sequence nucleotide, as well as the estimation of the evolutionary divergence between the sequences, were analyzed using DNA Baser (Dacia/P7, Mioveni 115400, Arges, Romania) and MEGA X software, respectively [21]. The same tool was used to perform a neighbor-joining analysis.

### Virus isolation

One milliliter of buffy coat cell suspension isolated from whole blood samples of each BVDV-positive goat was cultured and propagated in Madin–Darby bovine kidney (MDBK) cells. The cells were seeded for 24 h in 25-cm^2^ tissue culture flasks, and the viruses were allowed to adsorb to the MDBK cells for 1 h at 37°C. After incubation, the inoculum was removed, 6 mL of Eagle’s minimum essential medium containing Earle’s salt, l-glutamine, and sodium bicarbonate (M-4655 Sigma-Aldrich) supplemented with 2% BVDV-free fetal bovine sera, 1 mL of penicillin–streptomycin (Gibco, Billing, MT, USA) and 250 ug/mL amphotericin (Caisson Lab, USA) were then added to each flask. The flasks were incubated at 37°C in a 5% CO_2_ atmosphere for 4 days [22]. Cells were observed daily to check for possible CP effects of the virus.

After 4 days of incubation, the culture medium was withdrawn carefully using a Pasteur pipette. The MDBK cells were freeze–thawed 3 times, and the suspensions were centrifuged at 4000× *g* and 4°C for 30 min. The supernatant was used to inoculate MDBK cells seeded into a 96-well microtitration plate, which was incubated at 37°C in a 5% CO_2_ atmosphere for 4 days in a CO_2_ incubator. After incubation, the medium was removed, and the cells were washed 3 times with wash buffer (Phosphate buffered saline [PBS; pH 7.2] solution containing 0.05% Tween 20), drained, and fixed for 10 min at room temperature (26°C) using 100 mL of 35% acetone in a PBS solution containing 0.02% bovine serum albumin. After fixation, the BVDV-infected cells were visualized using the immunocytochemistry staining technique.

### Immunocytochemistry

Monoclonal antibody 15C5 (Idexx Laboratories, Westbrook, ME, USA) was used as the primary antibody in this study [23]. Fifty microliters of primary antibodies (1:100 dilution) were added to each well, followed by 45-60 min incubation at 26°C. After incubation, the remaining primary antibody was drained from the wells, and the cells were rinsed 3 times for 2 min each using 300 μL PBS. After washing, the wells were drained, and 100 μL of biotinylated secondary antibody was added. The cells were incubated for 30 min at 26°C. Subsequently, the wells were washed 3 times for 2 min each using 300 μL of PBS and 100 μL of conjugate enzyme streptavidin–horseradish peroxidase was added to each well. The plate was incubated for 10 min at 26°C. Finally, the wells were washed as previously described to remove the excess conjugate. For color development, 100 μL of diaminobenzidine chromogen was added to each well with the MDBK cells, and the plate was incubated for another 5-7 min. After incubation, the wells were drained, the cells were washed with sterile H_2_O once, and five drops of hematoxylin as a base dye were added, followed by incubation for 1 min. After incubation, the wells were drained, washed with sterile H_2_O, allowed to dry, and observed under the microscope.

## Results

**Figure-1:**
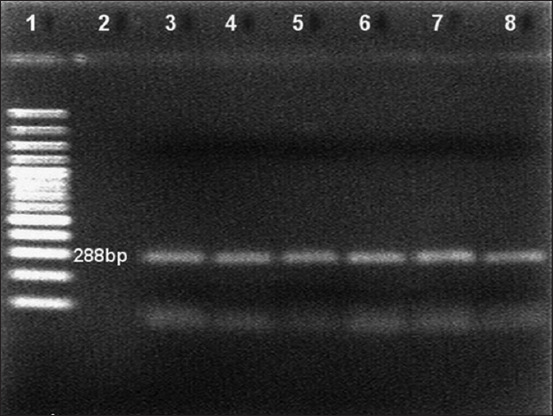
Reverse transcription-polymerase chain reaction analysis of samples for the 5’ untranslated region. Lane 1: 100 bp DNA marker, Lane 2: Negative control, Lane 3: Positive control and Lanes 4-8: Bovine viral diarrhea virus positive specimen.

**Figure-2:**
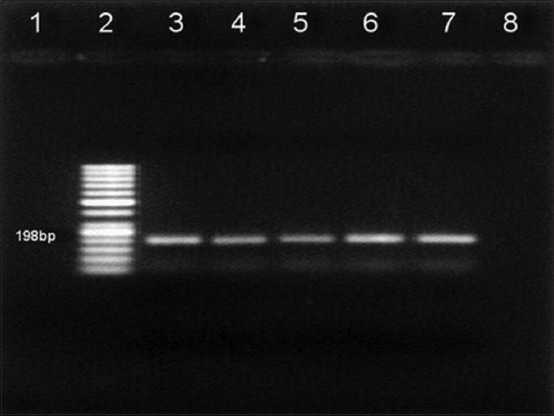
Reverse transcription-polymerase chain reaction test of samples for bovine viral diarrhea virus-1. Lane 1: Negative control, Lane 2: 50 bp DNA marker, and Lanes 3-7: Bovine viral diarrhea virus-1 positive specimen.

**Figure-3:**
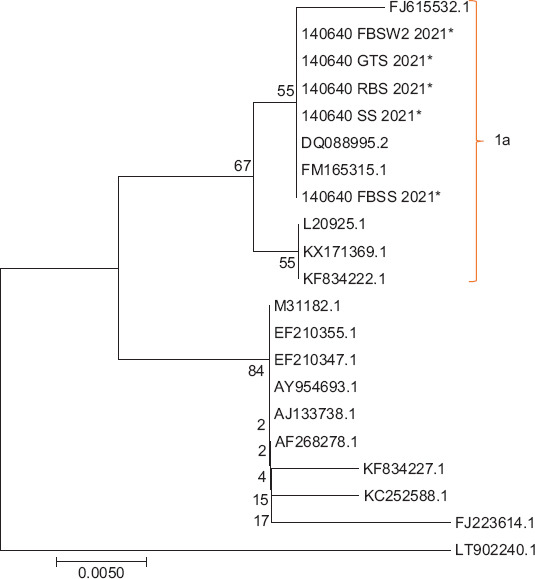
Phylogenetic tree based on a 288nt region of the 5’ untranslated region with the maximum likelihood method, general time reversible model, and 1000× bootstrap rate using MEGA X.

In this study, the clinical findings of dairy goats at the time of sampling were abortion (n=5), metritis (n=11), retention of the placenta (n=11), and repeated breeding (n=32). RNA extraction was done directly from sera. The RNA was amplified using a conventional polymerase chain reaction against the 5’ UTR of the gene having an expected size of 288 bp, and reactions were positive in five out of 39 samples (Figure-1). Antigen-positive samples were confirmed as BVDV-1 using BVDV-1-specific nested RT-PCR (Figure-2). Through phylogenetic and nucleotide sequence analyses of the 5’ UTR of the BVDV genome, sequences from all positive samples were clustered as BVDV-1a (Figure-3). All positive samples in this study were grouped in the clade with the representative subgenotype BVDV-1a FJ615532.1 sheep and the FM165315.1 buffalo isolate from Argentina [24]

**Figure-4:**
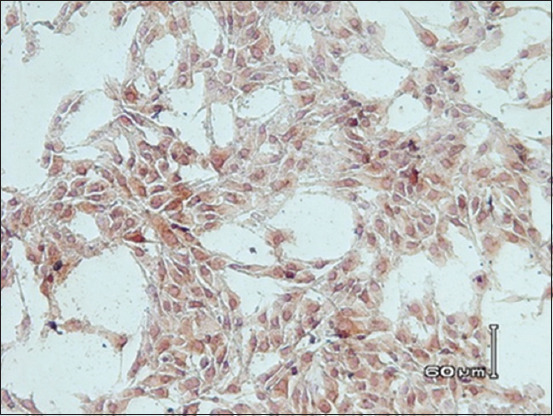
Image of Madin–Darby bovine kidney (MDBK) cells infected with noncytopathic bovine viral diarrhea virus (non-cytopathic [NCP] Bovine viral diarrhea virus [BVDV]). MDBK cells infected with NCP BVDV showed a brownish color in the nuclei and/or cytoplasm which was a deposit of NCP BVDV antigen (immunocytochemistry assay, 500×).

**Figure-5:**
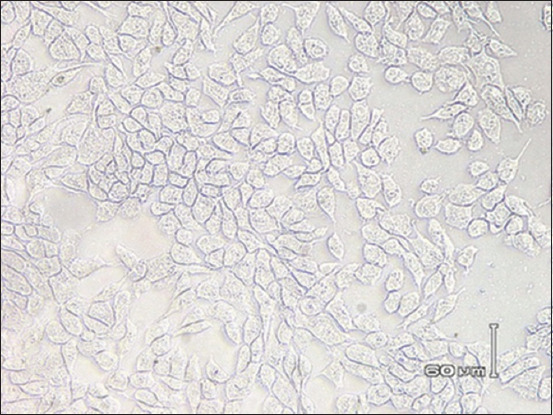
Image of Madin–Darby bovine kidney (MDBK) cells that were not infected with bovine viral diarrhea virus as examined by immunocytochemistry assay (ICC) *in vitro* (negative control). MDBK cells that appeared normal reached 80% monolayer confluence and exhibited no vacuolization and no color precipitation in the cytoplasm (ICC, 500×).

Figures-4 and 5 show the results of immunocytochemical staining. The brownish color in the nuclei and cytoplasm of the MDBK cells indicated that the BVDV in the samples was alive and replicating within the cells. However, the virus did not cause any cellular damage, suggesting that the biotype of BVDV was NCP.

## Discussion

Sapera is a crossbreed between an Etawah doe and a Saanen buck. In Yogyakarta, these goats are mainly raised and reared under smallholder management for milk production. Smallholder management can result in poor genetic quality because of uncertainty in the genetic potential of their parent goats [25]. Repeated breeding incidents are frequently reported in Sapera goats under smallholder management (personal communication). Therefore, only small farms that historically have low reproductive performance were included in this study to elucidate the possible relationships between low reproductive performance and the occurrence of BVDV infection.

In this study, for a period of 1 month, five cases of reproductive disorders that occurred naturally were reported from several flocks of Sapera dairy goats. The clinical status of the five goats that were molecularly positive for the presence of the BVDV antigen was as follows: Abortion (goat numbers 2 and 14), metritis (goat number 22), and repeated breeding without showing any clinical sign of disease (goat numbers 6 and 39). To the best of our knowledge, this is the first study to identify BVDV infection in Sapera dairy goats that have experienced natural reproductive failure in Indonesia. However, serological and molecular detection of BVDV infection has occurred in healthy local goats and sheep [16]. This study has shown that BVDV infection in Sapera dairy goats causes reproductive disorders. This finding is in agreement with previous reports [12,18]. BVDV infection (natural or experimental) in small ruminants during pregnancy can cause reproductive failures such as fetal resorption, fetal mummification, stillbirth, or abortion [11]. However, the clinical manifestations of BVDV infection during pregnancy in heterologous hosts are variable and depend on different host- and virus-associated factors but are generally characterized by reproductive disorders such as poor conception, intrauterine infection, abortion, and poor neonate viability [26-28].

Experimentally, infection of pregnant goats with BVDV-1 or BVDV-2 leads to considerably different outcomes. Infection with BVDV-2 can induce severe reproductive disease, as is common for BVDV infection in goats. In contrast, infection with BVDV-1 is clinically less apparent and could disseminate the virus [18]. However, field observations indicate differently; the same manifestation of the disease can occur in both BVDV-1 and BVDV-2 infections [29].

In early genetic characterization, the genome of BVDV was classified into two different genetic species, namely, BVDV-1 and BVDV-2. Of the two species, BVDV-2 has been associated with severe hemorrhagic disease, which resulted in high mortality in Canada, whereas BVDV-1 is the most common cause of asymptomatic infection [30]. A third genetic species, BVDV type 3, described as atypical BVDV, has been reported [31] but officially remains unrecognized [32]. Based on the phylogenetic analysis of partial sequences from the 5’ UTR, Npro, glycoprotein E2, NS3, and NS5B-3’ UTR, BVDV-1 in cattle can be further differentiated into 21 genetic subtypes (BVDV-1a to BVDV-1u) [33,34] and BVDV-2 into four genetic subtypes (BVDV-2a – BVDV-2d) [35,36]. The genetic variability of BVDV-1 in goats was first reported in southwestern China. Five different subtypes, namely, BVDV-1a, BVDV-1b, BVDV-1c, BVDV- 1m, and BVDV-1p, were identified with BVDV-1b and BVDV-1m as the main subtypes in Chinese goat herds [37].

Based on phylogenetic and nucleotide sequence analyses from three different BVDV genomic regions (5’ UTR, NS5B, and Erns), the most prevalent BVDV group in Indonesian cattle is BVDV-1a, whereas BVDV-1b, BVDV-1c, and BVDV-1d are less prevalent [38]. In the present study, using BVDV-1-specific nested RT-PCR, antigen-positive samples were confirmed to be BVDV-1. BVDV-2 was not detected in any of these samples. All of the sequences from our positive samples were clustered within BVDV-1a, had 99% homology to each other, and shared high similarity (90% homology) with the BVDV-1a reference strains Singer (DQ088995.2), isolate FJ615532.1 circulating in sheep in Argentina, and isolate FM165315.1 in buffalo in Argentina [24]. Although the genetic variability was not determined, infection with BVDV-1 has also been reported in healthy local Indonesian goats [16]. Therefore, based on the research results, it could be assumed that BVDV-1a is a major contributor to BVDV infection in cattle or goats in Indonesia. However, to determine the prevalence and further our understanding of the genetic diversity of BVDV-1 in Indonesian goats, research that involves a more representative number of BVDV-1 positive samples needs to be conducted in the future. The genetic diversity of BVDV has serious clinical implications such as immune evasion, increased virus virulence, and host range alteration, which affects the efficacy of vaccination programs and diagnostic methods [39,40].

The most important source of BVDV infection in cattle is the apparently healthy PI virus carrier. However, persistent infection generally does not occur naturally in goats but has been reported in experimental cases [18]. In the present study, the primary source of BVDV infection in Sapera dairy goats was unclear because the goats did not share cages or grazing areas with cattle or sheep. However, because the goats used were from smallholder farmers, trade movement for either renewal of breeding stock to increase herd size or provide cash may represent a method of spreading the pathogen. In this study, we did not check the persistence of BVDV infection in the herds because of limited research time.

Based on virus isolation, all of the antigen-positive samples in the present study had an NCP biotype (NCP-BVDV), which is in agreement with that of previous reports [40]. Approximately 90% of the field isolates of BVDV have been reported to be the NCP biotype [8], which is of particular clinical importance because it is capable of crossing the placenta of an acutely infected dam [41]. Mutation of the NCP strain can give rise to the CP biotype in animals, resulting in the development of mucosal disease.

## Conclusion

The results of our present study provide valuable data on the clinical manifestations caused by BVDV-1a infection in Sapera dairy goats in Sleman Yogyakarta, and they showed that BVDV-1a is the main subtype present in the population. These results can facilitate the evaluation of methods for the prevention and control of BVD in Indonesia. However, for better evaluation of the interrelationships between BVDV infection and reproductive disorders in Sapera dairy goats, future studies are needed with more samples and a longer study period.

## Authors’ Contributions

NR: Blood sampling, RNA extraction, and RT-PCR analysis. HW: Tissue culture and immunocytochemistry. SHI: Sequencing and phylogenetic analyses. HW and RW: Supervised the experiments. All authors participated equally in preparing the manuscript for publication. All authors read and approved the final manuscript.
